# Risk of Recurrence in Laryngeal Cancer

**DOI:** 10.1371/journal.pone.0164068

**Published:** 2016-10-07

**Authors:** Jesper Brandstorp-Boesen, Ragnhild Sørum Falk, Jan Folkvard Evensen, Morten Boysen, Kjell Brøndbo

**Affiliations:** 1 Department of Otorhinolaryngology, Division of Surgery and Clinical Neuroscience, Oslo University Hospital, Oslo, Norway; 2 Oslo Centre for Biostatistics and Epidemiology, Research Support Service, Oslo University Hospital, Oslo, Norway; 3 Department of Oncology, Oslo University Hospital, Radiumhospitalet, Oslo, Norway; 4 University of Oslo, Institute of Clinical Medicine, Oslo, Norway; University of Cincinnati College of Medicine, UNITED STATES

## Abstract

A cohort study was undertaken to analyze the risk of recurrence among 1616 patients with primary squamous cell carcinoma of the larynx from 1983 to 2010 at a single, tertiary academic center in Oslo, Norway. The cohort was followed from the date of diagnosis to September 2011. Competing risk regression analysis assessed the association between various risk factors and the risk of recurrence, where death was considered a competing event. Recurrence was observed in 368 patients (23%) during the study period. The majority (71%) of recurrences involved the location of the primary tumor. The overall risk of recurrence during the first three years after initiating treatment was 20.5%. Increased risk of recurrence was observed in patients with supraglottic cancer, younger patients, those with T2–T3 tumors and in patients treated in the earlier part of the study period. Significant factors for recurrence in glottic carcinomas were age, treatment in the earlier part of the study and T-status, whereas age was a significant factor in supraglottic cancer. N-status appeared less significant. In conclusion, follow-up of laryngeal squamous cell carcinoma should place particular emphasis on the site of the primary tumor, younger patients, cases of supraglottic cancer and T2-T4 primary tumors, especially during the first three years after treatment. More studies are needed to assess the impact of surgical versus non-surgical treatment, and eventually the significance of recurrence, for disease-specific and overall survival in cases of advanced laryngeal squamous cell carcinoma.

## Introduction

Laryngeal squamous cell carcinoma (LSCC) accounts for approximately 17% of all primary head and neck cancers (HNSCC) in Norway, with an age-standardized incidence rate (ASR) for laryngeal cancer of 1.4 per 100,000 in 2014 [1]. Males with glottic cancer predominate, but during the last three decades the proportion of females has increased significantly [2].

The treatment of early and advanced stage laryngeal cancer has been subject to a substantial development during the last three decades. The objective of LSCC management is cure with preserved laryngeal function. To achieve this, it is vital to assess the risk of recurrent disease in each case. The risk of recurrence varies considerably with the modality of treatment, as with subsite, N-status and T-status [3, 4]. Early stage laryngeal cancer is generally associated with high local control rates and a favorable outcome [5]. However the recurrence rates of advanced stage LSCC have been reported to range between 25–50% [6]. Moreover, previous studies have shown that laryngeal recurrences primarily develop in the region of the primary tumor and within three years after the primary therapy [7].

Early detection of recurrent disease in LSCC is an important contributor to a successful disease outcome [8]. Thus, identification of prognostic factors for recurrence would be highly relevant to the clinician. The objective of this study was to analyze the subsite-specific risk factors for recurrence in patients treated for LSCC.

## Materials and Methods

All patients diagnosed with primary LSCC at the Department of Otorhinolaryngology, Oslo University Hospital, Rikshospitalet during 1983–2010 were included in the study. The Privacy and Data Protection Office, CEO Executive Staff, Oslo University Hospital approved the study and data collection was authorized by the Norwegian Data Protection Authority. Written consent was collected from each patient at the time of diagnosis. Baseline data on gender, smoking/alcohol status, age, subsite and TNM status at diagnosis as well as date of diagnosis, date of primary treatment, modality of treatment and follow-up were obtained from the hospital records and compiled in a database. Information about deaths (or date of emigration) was obtained from the hospital patient registration system, which is updated regularly from the Cause of Death Registry. The data were linked through unique personal identification numbers, which are assigned to every individual in Norway. Longitudinal follow-up of the cohort was continued until 30 September 2011. The Ministry of Health approved the study and data collection was authorized by the Norwegian Data Inspectorate.

After clinical and radiological examination, all patients were evaluated by a multidisciplinary tumor board of head and neck surgeons and oncologists from the Norwegian Radium Hospital. The final management was performed in accordance with the Danish Head and Neck Cancer Group (DAHANCA) guidelines, which have been applied since 1995 [9]. Between 1983–1995, T1AN0M0 glottic carcinomas were treated with conventional RT (66 Gy, 2 Gy per fraction, 5 fractions/week), but since 1 January 1996, TLM has been the standard treatment for these tumors. From 1983, early and intermediate stage laryngeal cancer was managed by a conventional RT scheme (68–70 Gy, 5 fractions/week) but during1995–2000 an accelerated protocol (6 fractions/week) was gradually introduced according to DAHANCA guidelines [10]. Concomitant CRT was introduced as standard treatment for advanced stages in 2002, whereas T4a laryngeal cancers continued to be treated with TLAR and post-operative RT (50 Gy). Partial laryngectomy is not part of standard treatment at our center and has only been performed in a few selected cases.

Regardless of stage and treatment modality, all patients were evaluated clinically 4–6 weeks after primary treatment. Before 1990, endoscopy was performed with a mirror or Hopkins rod and after 1990 by means of flexible videolaryngoscopy, supplemented with stroboscopy as appropriate. A computed tomographic (CT) scan of the neck was performed before the first consultation for all patients (except T1a glottic cancer treated by TLM) and was repeated annually and/or when required for a complete assessment. Initially, conventional chest x-ray was performed regularly but was stopped halfway through the study due to the low detection rate for metastases and secondary malignant tumors. Ultrasound examination of the neck with fine-needle cytology and/or micro-laryngoscopy with a biopsy was performed when required. Patients were seen every 8–12 weeks during the first year and 2–3 times during the second and third years post-treatment. Thereafter the surveillance was continued by the local Ear, Nose and Throat Department in the majority of cases. Any suspicion of recurrence during follow-up led to immediate referral to our institution for further examination and treatment.

As per the department protocol, first follow-up consultations were carried out by the senior staff member, who was responsible for the initial TNM classification and the primary treatment decision. Our database is updated continuously regarding the site of recurrence and occurrence of death. The date of recurrence was defined at the date of histological verification. Recurrences were categorized as local, regional, loco-regional or metastases at distant sites. In case of simultaneous recurrence in more than one site, both sites were registered. Recurrence in the stoma in patients subjected to primary total laryngectomy was defined as local-stoma.

The cohort was categorized by gender, smoking/alcohol (ever, never, unknown), age (≤59, 60–69 and ≥70 years), subsite (glottic, supraglottic or subglottic) and stage of disease. All tumors were classified in accordance with the UICC TNM staging system and the AJCC TNM (stage I–IV), where early stage (I+II) is defined as T1-T2N0M0, and advanced stage (III+IV) is defined as T3–T4a/b and any TN+, M+. The cohort was divided into four time periods according to the year of initial management (1983–1989, 1990–1996, 1997–2003, 2004–2010) and categorized by one of the treatment modalities: RT, TLM, TLAR, CRT or palliative/no treatment.

### Statistics

Descriptive statistics are presented as frequencies and proportions. The cohort was followed up longitudinally from the date of primary diagnosis, whereas the date of initiated treatment was applied as start point in the risk analysis. Due to the possibility of death during the follow-up period, death as a competing event was incorporated into the analysis. Thus, the patients were followed to the date of histologically verified recurrence or censored at the date of study closure (30 September 2011), or considered as a competing event at the time of death (from any cause), whichever occurred first. The cumulative risk of recurrence, which describes the absolute risk over the time course, was plotted during 10 years of follow-up and is presented as risk estimates at three years after treatment of LSCC. The Pepe and Mori test was performed to compare the cumulative risk of glottic versus supraglottic cancer [11]. Univariate and multivariate competing risk analyses, using the model of Fine and Gray [12], were performed to evaluate the effect of potential risk factors for recurrence during 10 years of follow-up after treatment of LSCC. Stage could not be included in the model due to high correlation with T- and N-status. Smoking/alcohol was omitted from the multivariate model due to lack of detailed data on consumption. Risk estimates are presented as sub-distribution hazard ratios (SHR) with accompanying 95% confidence intervals (CI) and p values. The analyses are stratified by subsite to meet the model assumptions of proportional hazards. Only glottic and supraglottic carcinomas had a sufficient number of patients for analysis. Sensitivity analyses were conducted by restricting the follow-up to three years after initiation of treatment, due to the low number of cases followed beyond three years (11%).

P-values ≤0.05 were regarded as statistically significant. Data analysis was performed using SPSS [13] and Stata [14].

## Results

In total, 1616 patients were diagnosed with primary LSCC during the study period. One patient was excluded since he died on the day of diagnosis, such that 1615 patients were included in the analysis. The long-term descriptive trends of the study cohort have been published previously [2].

Among these 1615 cases, 368 (23%) patients developed recurrent disease. Death as a competing event occurred in 674 (42%) patients. The median follow-up time for the whole cohort was 3.2 years (range 0–28.3 years). Patients with and without recurrent disease had a median follow-up of 1.0 years and 5.2 years, respectively. Ninety-eight percent of patients (n = 1583) were treated with curative intent, while 2% (n = 32) were considered medically or mentally unfit for curative treatment or the patients abstained from treatment.

**Fig 1:**
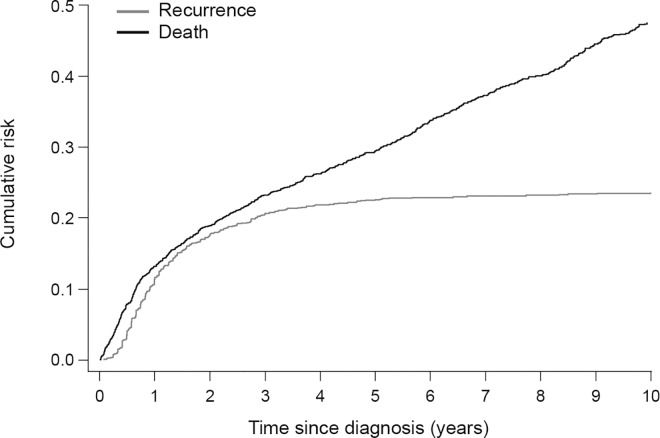
Cumulative risk of recurrence and death during 10 years' follow-up among patients with laryngeal squamous cell carcinoma.

**Fig 2:**
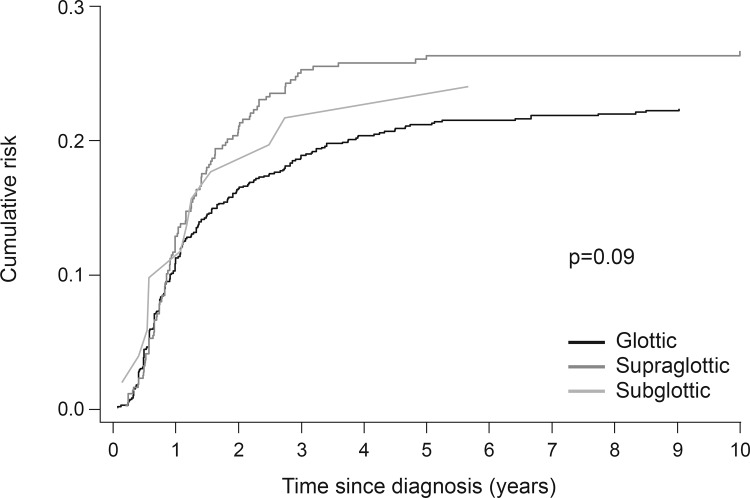
Cumulative risk of recurrence by subsite of laryngeal squamous cell carcinoma during 10 years' follow-up. P value is achieved from the Pepe and Mori test comparing the cumulative risk of glottic versus supraglottic cancer.

Patient and disease characteristics are summarized in Table 1. In the first period (1983–89) the risk of recurrence was 28% (113/402), after which the risk declined gradually to 17% (73/434) in the last period (2004–10). The cumulative risk of recurrence for the whole cohort at 1, 3, 5 and 10 years of follow-up was 11.3%, 20.5%, 22.5% and 23.6%, respectively, while the risk of death increased steadily over time (Fig 1). The risk of recurrence tended to be more striking in supraglottic versus glottic carcinomas over the 10 years following treatment, although this was not statistically significant (p = 0.09, Fig 2).The three-year risk of recurrence decreased by age at diagnosis and period of treatment (Table 2). Furthermore, the three-year risk was lowest for T1 (11%) and T4 (21.1%) laryngeal cancer, while T2 (27.3%) and particularly T3 (35.8%) laryngeal cancer were associated with higher risks for recurrence. The risk of recurrence increased with increasing nodal involvement and stage (early versus advanced) (Table 2). Among patients with T1a glottic cancer, the risk of recurrence was similar in patients treated with RT (three-year cumulative risk 8.7%, 95%CI 5.6–12.7%) or TLM (8.7%, 5.9–12.3%).

**Table 1:** Patient and disease characteristics and number of recurrences during 10 years' follow-up of 1615 patients with laryngeal squamous cell carcinoma treated during 1983–2010.

	Recurrence n = 368		Non-recurrence n = 1247	
	n	%	n	%
**Sex**				
Male	322	88	1081	87
Female	46	12	166	13
**Smoking**				
Ever	326	89	1108	89
Never	30	8	67	5
Unknown	12	3	72	6
**Alcohol**				
Ever	61	17	162	10
Never	213	58	715	60
Unknown	94	25	370	30
**Age (years)**				
0–59	130	35	328	26
60–69	131	36	411	33
≥70	107	29	508	41
**Subsite**				
Glottic	243	66	884	71
Supraglottic	113	31	324	26
Subglottic	12	3	39	3
**T-status**				
T1	90	24	577	46
T2	120	33	267	21
T3	85	23	146	12
T4	73	20	257	21
**N-status**				
N0	300	82	1043	84
N1	23	6	76	6
N2+	45	12	128	10
**M-status**				
M0	368	100	1233	99
M1	0	0	14	1
**Stage**				
Early stage	194	53	789	63
Advanced stage	174	47	458	37
**Stage I-IV**				
I	87	24	563	45
II	107	29	223	18
III	76	21	146	12
IV	98	26	315	25
**Treatment modality**				
Radiotherapy	271	74	737	59
Transoral lasermicrosurgery	38	10	294	24
Total laryngectomy	41	11	138	11
Chemo-radiotherapy	18	5	37	3
Palliative/ no treatment	0	0	41	3
**Period of treatment**				
1983–1989	113	31	289	23
1990–1996	89	24	288	23
1997–2003	93	25	309	25
2004–2010	73	20	361	29
**Length of follow-up**				
<1 year	183	50	222	18
1–2 years	143	39	239	19
3–5 years	34	9	237	19
6–10 years	8	2	549	44

**Table 2:** Cumulative risk of recurrence by patient and disease characteristics at 3 years' follow-up among patients with laryngeal squamous cell carcinoma.

	n	3-year risk	95% Confidence interval	
**Overall**	326	20.5	18.6	22.5
**Sex**				
Male	283	20.5	18.4	22.6
Female	42	20.8	15.5	26.5
**Smoking**				
Ever	295	21.1	19.0	23.3
Never	22	24.3	16.2	33.4
Unknown	9	12.0	6.2	20.0
**Alcohol**				
Ever	57	26.3	20.7	32.2
Unknown	77	17.1	13.8	20.7
**Age (years)**				
0–59	114	25.2	21.3	29.3
60–69	121	22.7	19.2	26.3
70+	91	15.1	12.3	18.1
**Subsite**				
Glottic	208	18.7	16.5	21.1
Supraglottic	107	25.1	21.0	29.3
Subglottic	11	21.7	11.6	33.8
**T-status**				
T1	72	11.0	8.8	13.6
T2	104	27.3	22.9	31.9
T3	81	35.8	29.6	42.1
T4	69	21.1	16.9	25.7
**N-status**				
N0	260	19.7	17.6	21.9
N1	23	23.4	15.6	32.1
N2+	43	25.5	19.2	32.3
**Stage**				
Early stage	161	16.7	14.4	19.1
Advanced stage	165	26.6	23.2	30.1
**Stage I-IV**				
I	69	10.9	8.6	13.4
II	92	28.2	23.4	33.1
III	73	33.6	27.4	39.9
IV	92	22.6	18.7	26.8
**Treatment modality**				
Radiotherapy	238	23.8	21.2	26.5
Transoral lasermicrosurgery	31	9.6	6.7	13.1
Total laryngectomy	39	21.9	16.2	28.3
Chemo-radiotherapy	18	36.6	23.4	49.9
**Period of treatment**				
1983–1989	103	25.6	21.5	30.0
1990–1996	75	19.9	16.0	24.1
1997–2003	80	19.9	16.2	23.9
2004–2010	68	16.8	13.3	20.7

When a competing risk regression model was used to study the association between potential factors and the risk of recurrence in glottic cancer (Table 3), the risk decreased significantly for patients treated in the last period compared to the first period (SHR 0.5, 95% CI 0.3–0.8). The risk of recurrence was significantly lower for patients aged ≥70 years compared to <60 years (SHR 0.6, 95% CI 0.5–0.9), and in patients with T1a carcinomas compared to T1b-T4 carcinomas (Table 3). Involvement of two or more regional neck nodes increased the risk of recurrence in glottic cancer but not significantly (SHR 1.5, 95% CI 0.9–2.7). The results of univariate and multivariate analysis were of the same magnitude except for TLM and T3-4, which increased the risk of recurrence in multivariate analysis.

**Table 3:** Competing risk regression model to evaluate the effect of selected covariates on the risk of recurrence among patients with glottic laryngeal squamous cell carcinoma during 10 years' follow-up (n = 1127; 243 recurrences and 425 deaths). SHR, sub-distribution hazard ratios; CI, confidence interval.

	Univariate analysis		Multivariate analysis	
	SHR (95% CI)	P value	SHR (95% CI)	P value
**Sex**				
Male	1		1	
Female	0.67 (0.40–1.11)	0.12	0.66 (0.39–1.13)	0.13
**Age (years)**				
0–59	1		1	
60–69	0.79 (0.59–1.07)	0.12	0.83 (0.61–1.12)	0.23
70+	0.57 (0.42–0.79)	<0.01	0.62 (0.45–0.85)	<0.01
**T-status**				
T1a	1		1	
T1b	3.46 (1.99–6.01)	<0.001	3.93 (2.14–7.21)	<0.001
T2	3.52 (2.54–4.88)	<0.001	4.04 (2.65–6.17)	<0.001
T3	4.39 (3.03–6.37)	<0.001	5.79 (3.55–9.44)	<0.001
T4	1.88 (1.21–2.93)	<0.01	2.67 (1.39–5.12)	<0.01
**N-status**				
N0	1		1	
N1	1.12 (0.56–2.24)	0.74	0.90 (0.43–1.87)	0.77
N2+	1.65 (0.96–2.85)	0.07	1.52 (0.85–2.72)	0.16
**Stage I-IV**				
I	1			
II	3.06 (2.25–4.18)	<0.001		
III	3.54 (2.46–5.10)	<0.001		
IV	1.81 (1.22–2.69)	<0.01		
**Treatment modality**				
Radiotherapy	1		1	
Transoral lasermicrosurgery	0.43 (0.30–0.61)	<0.001	1.58 (0.93–2.71)	0.09
Total laryngectomy	0.85 (0.55–1.31)	0.46	0.79 (0.45–1.40)	0.43
Chemo-radiotherapy	2.14 (1.07–4.31)	0.03	1.92 (0.89–4.16)	0.10
**Period of treatment**				
1983–1989	1		1	
1990–1996	0.79 (0.56–1.11)	0.18	0.80 (0.57–1.15)	0.23
1997–2003	0.75 (0.53–1.04)	0.09	0.68 (0.47–1.00)	0.05
2004–2010	0.59 (0.41–0.85)	<0.01	0.50 (0.33–0.77)	<0.01

Table 4 present the risk of recurrence in supraglottic cancer. Patients aged ≥70 years had a significantly lower risk of recurrence than patients aged <60 years (SHR 0.6, 95%CI 0.4–0.9). In contrast to glottic cancer, female gender showed a tendency to increase the risk of recurrence in supraglottic cancer compared to males (p = 0.08). Nodal involvement did not increase the risk of recurrence among supraglottic cancer patients. Unlike the case in glottic cancer, there was no significant difference in the risk of recurrence between study periods.

**Table 4:** Competing risk regression model to evaluate the effect of selected covariates on the risk of recurrence among patients with supraglottic laryngeal squamous cell carcinoma during 10 years' follow-up (n = 437; 113 recurrences and 227 deaths). SHR, sub-distribution hazard ratios; CI, confidence interval.

	Univariate analysis		Multivariate analysis	
	SHR (95% CI)	P-value	SHR (95% CI)	P-value
**Sex**				
Male	1		1	
Female	1.36 (0.90–2.07)	0.14	1.46 (0.95–2.24)	0.08
**Age (years)**				
0–59	1		1	
60–69	0.97 (0.63–1.50)	0.90	1.02 (0.65–1.59)	0.94
70+	0.56 (0.35–0.89)	0.01	0.58 (0.37–0.92)	0.02
**T-status**				
T1	1		1	
T2	1.34 (0.71–2.53)	0.37	1.19 (0.61–2.29)	0.61
T3	2.02 (1.06–3.82)	0.03	1.67 (0.86–3.24)	0.13
T4	1.24 (0.67–2.31)	0.50	1.20 (0.61–2.38)	0.60
**N-status**				
N0	1		1	
N1	0.79 (0.44–1.41)	0.42	0.73 (0.40–1.33)	0.30
N2+	0.88 (0.57–1.35)	0.56	0.91 (0.56–1.49)	0.70
**Stage I-IV**				
I	1			
II	1.46 (0.70–3.04)	0.31		
III	1.72 (0.84–3.50)	0.14		
IV	1.34 (0.70–2.57)	0.38		
**Treatment modality**				
Radiotherapy	1		1	
Total laryngectomy	0.75 (0.42–1.33)	0.33	0.69 (0.37–1.29)	0.25
Chemo-radiotherapy	0.97 (0.53–1.77)	0.91	0.88 (0.46–1.66)	0.69
**Period of treatment**				
1983–1989	1		1	
1990–1996	0.85 (0.51–1.40)	0.51	0.82 (0.48–1.40)	0.46
1997–2003	0.87 (0.52–1.45)	0.60	0.90 (0.52–1.58)	0.72
2004–2010	0.62 (0.36–1.08)	0.09	0.63 (0.33–1.21)	0.16

Sensitivity analyses restricted to three years of follow-up gave similar results (SHRs) to those observed from the full follow-up analyses (data not shown).

The majority (71%) of recurrences involved only the site of the primary tumor (Table 5). The three-year cumulative risks for local and regional recurrence were 14.1% and 3.1%, respectively. Distant recurrences were relatively rare, and most often of pulmonary origin (6%). The site of recurrence differed significantly between subsite (p<0.01) (Table 4). Local recurrence predominated for all subsites, but regional relapses were more frequent among supraglottic carcinomas. Among patients treated with TLAR, the three-year cumulative risk of stoma recurrence was 6.7% and primarily related to recurrence of a glottic cancer.

**Table 5:** Type of recurrence stratified by subsite at diagnosis. Chi-square test, used to determine whether the site of recurrence differed between subsites, yielded p<0.01.

Type of recurrence	Overall		Glottic		Supraglotttic		Subglottic	
	n	%	n	%	n	%	n	%
Local	261	70.9	190	78.2	64	56.7	7	58.4
Regional	50	13.6	25	10.3	24	21.2	1	8.3
Loco-regional	13	3.5	7	2.9	6	5.3	0	0
Local stoma	13	3.5	9	3.7	3	2.7	1	8.3
Distant pulmonary	22	6.0	8	3.3	12	10.6	2	16.7
Loco-regional+ distant	9	2.5	4	1.6	4	3.5	1	8.3
**Total**	368		243		113		12	

## Discussion

This study of 1615 LSCC patients represents approximately 60% of all patients diagnosed with LSCC in Norway between 1983 and 2010. Slightly less than one-quarter of our cohort developed recurrent laryngeal cancer during 10 years of follow-up, corresponding to findings in the literature [15, 16]. Recurrent disease occurred early and predominantly at the site of the primary tumor, and the risk of recurrence was associated with age, subsite, stage and the modality of treatment.

Encouragingly, we observed a significant drop in the rate of glottic LSCC recurrence during the more recent study periods. This is probably due to several factors, but improvement in the initial staging and hence the choice of primary management is likely to have contributed. Moreover, since this improvement only applied to glottic cancer, recognition of early onset symptoms (hoarseness) may have had an impact on the course of the disease. The information on smoking and alcohol use prior to the diagnosis of LSCC, during treatment or follow-up in our cohort, could only be presented as ever, never or unknown. Nevertheless, as the number of daily smokers in Norway has decreased from 42% in 1973 to 13% in 2014, smoking cessation could be a possible confounder to our findings.

Outcomes in patients with HNSCC have been studied extensively at our institution and elsewhere [17]. Consistent with the studies of Boysen *et al* [7] and Kothari *et al* [18], we have demonstrated the importance of clinical follow-up for at least three years, since almost 90% of LSCC recurrences observed over a 10-year period were confirmed by year 3 after the beginning of treatment. The fact that 50% of our patients experienced relapse within 12 months strongly supports the need for frequent follow-up (every 6–8 weeks) and exclusively at high-volume specialist centers, at least for the first year. Intensive follow-up is also more likely to reduce the risk that the patients will ignore symptoms which indicate recurrence, thereby improving compliance [19]. We recommend that the senior laryngologist, or the head and neck surgeon responsible for the initial classification and management, performs the early follow-up consultations and subsequently supervises post-treatment examinations. Moreover, we acknowledge the necessity of individualized follow-up regimens for a subgroup of patients, as identified by Lester and Wight [20].

Regardless of subsite, most recurrences were found to develop locally. Supraglottic cancer presented more often with regional involvement both at diagnosis and at recurrence in our material. Nevertheless positive N-status at diagnosis did not increase the risk of recurrence in supraglottic cancer, as it did among glottic cancer. Of the 368 patients with recurrent disease, 271 (74%) were managed by RT as primary treatment. Endoscopic evaluation of an irradiated larynx, with local edema, fibrosis and necrosis of the mucosa and cartilage, is a well-known challenge [21]. Repeated endoscopic, radiological and histological procedures are non-specific examinations, and residual tumor manifestations or recurrent disease may be overlooked [15, 22]. The fact that the biopsy itself can aggravate post-RT conditions is well-recognized. Although CT or MRI examinations are frequently inconclusive with regard to residual tumor or loco-regional recurrence, we have thus far relied on direct laryngoscopy and a CT scan of the neck. Use of a 18F-FDG-PET scan to verify loco-regional recurrent LSCC has shown promising results and may prove a more accurate modality [23, 24, 25].

There were significant differences in the cumulative incidence of recurrence according to T-status, confirmed by multivariate analysis, with T2 and T3 LSCC being the least favorable. Several studies have pointed out the heterogeneous nature of T2 glottic cancer [26, 27]. A SEER-based study by Chen *et al* [28], and a follow-up study from the Netherlands [29], has shown similar results regarding the impact of T2 tumors on local control. In both reports, the authors sub-classified T2 glottic cancer into T2a (preserved vocal cord mobility) and T2b (impaired vocal cord mobility), and highlighted the negative impact T2b tumors may have on outcome. We do not differentiate between T2a and T2b glottic tumors but cannot discount a possible unfavorable impact of T2b tumors on the risk of recurrence in our population. In a study by Haapaniemi *et al* [30] about laryngeal cancer in Finland, T2 glottic and T2 supraglottic cancer showed unexpectedly inferior disease-specific survival. The authors had no clear explanation for this outcome, but misclassification between T2-T3 tumors and lack of surgical intervention during management were proposed as possibilities. Although the results from Finland were based on Kaplan-Meier estimates, the high proportion of disease relapses among T2-T3 glottic carcinomas is in line with our results. We agree with Chen *et al* that future studies should strive to improve treatment of T2 glottic cancer.

Adoption of TLM as the standard treatment for T1a glottic cancer in 1996 (approximately midway through the study) coincided with a gradual increase in the proportion of early stage glottic cancer, classified here as T1a glottic carcinomas [2]. Early stage (T1a) glottic cancer was associated with a lower risk of recurrence. We found no difference in risk when comparing the cumulative incidence of recurrent T1a glottic cancer treated with RT (before 1996) or TLM (after 1996). This is consistent with findings from the study from Finland [30]. Moreover, it corresponds with the difference in effect of TLM on the risk of recurrence shown in our uni- and multivariate analyses. Nevertheless, we support the view put forward by Jäckel *et al* [31] and others [32, 33], that primary TLM offer the possibility of re-resection, after which there should be close follow-up. In addition, by using TLM as primary intervention, RT is kept in reserve as a salvage option. Further studies must clarify the role of TLM as primary intervention for intermediate and advanced LSCC as well as the salvage rates after TLM re-resection of recurrent glotttic carcinomas.

At our center, concomitant CRT has been part of the standard treatment for advanced LSCC (T1-2N+, T3, T4b) since 2002, but TLAR is still regarded as the primary approach for T4a tumors. Of the 179 TLAR patients in this cohort, less than one-fifth developed recurrent disease. Recurrence primarily involved the stoma or the regional neck nodes, to the same extent. The majority of the T3 tumors received either RT or CRT as primary combined treatment and primary TLAR was performed only in the event of tumor growth through the thyroid cartilage (T4a). In a recent study by Elegbede *et al* [34], non-surgical and surgical treatment of advanced supraglottic cancer was compared. Despite preservation of the larynx and similar overall survival, non-surgical treatment of advanced supraglottic cancer was associated with a higher rate of recurrence. Nguyen-Tan *et al* have reported promising results which favor surgery (TLAR and supraglottic laryngectomy) for achieving loco-regional control in advanced T3-T4 glottic and supraglottic LSCC [35]. This is supported by a survey conducted over three decades by Rosenthal *et al* concerning the long-term surgical and non-surgical outcomes for T4 LSCC [36]. Due to the relatively small number of cases treated with concomitant CRT in our study, comparisons of surgical and non-surgical management of advanced LSCC should be treated with caution, but calls for future studies. Regarding partial laryngectomy, alone or in combination with radiotherapy as primary treatment for advanced LSCC, it has only been applied exceptionally at our department. However, primary as well as salvage partial laryngectomy it is a well adapted procedure in line with TLAR in many institutions worldwide [37, 38].

Women in our cohort presented more often with a primary supraglottic carcinoma [2]. Among females with recurrent disease, two-thirds were treated for an advanced supraglottic carcinoma. In glottic cancer, female gender tended to decrease the risk of recurrence compared to men (SHR 0.66), while in supraglottic cancer female gender seemed to increase the risk of recurrence (SHR 1.46). Despite the non-significant nature of this effect of gender, the actual difference in the SHR-estimates between glottic and supraglottic cancer makes this finding interesting. Moreover, the fact that supraglottic cancer appeared more prone to recur than glottic cancer in our study, the possible association between female gender and subsite require closer scrutiny during future follow-up.

A possible disadvantage by our study could lie in its retrospective nature and the potential of inadequate information. This was unfortunately the case with respect to detailed smoking and alcohol consumption data. However, we have no indications that recurrences have been missed but the risk of delay or lack of verification of recurrences is present as some patients were transferred for further follow-up outside our institution. To avoid such pitfalls, we emphasize our very close cooperation with the referring entities during follow-up. The strength of our study lies in the large cohort and the uniform clinical investigation and treatment. Additionally, the absolute and relative risk of recurrence was analyzed by both cumulative risk estimates and competing risk regression analysis. The advantage of competing risk analysis, compared to the widely used Kaplan-Meier method, is that competing risk of death during follow-up is incorporated into the assessment of the impact of risk factors of recurrence [39].

In conclusion, recurrent laryngeal cancer developed locally and predominantly within the first three years of follow-up. The significant reduction in number of recurrences during the latter part of the study is encouraging but intermediate T-status (T2/T3) was surprisingly unfavorable regarding the risk of recurrence. Positive N-status increased the risk of recurrence among patients with glottic cancer but had an unexpectedly low impact on risk among supraglottic cancer patients. Older age (>70 years) decreased the risk of recurrence significantly in both glottic and supraglottic cancer, whereas female gender seemed to increase the risk only when treated for a supraglottic cancer. Moreover, supraglottic LSCC presented more frequently with recurrence than glottic LSCC and often with a loco-regional involvement, which is why this subgroup should be carefully monitored. Whether these findings indicate that the management of supraglottic cancer and T2-T3 tumors should be intensified is not yet clear. The low risk of recurrence was equivalent for T1a glottic cancers treated with RT or TLM and ongoing studies are evaluating a potential role for TLM as a salvage option. More studies are needed to assess the impact of surgical versus non-surgical treatment, and eventually the significance of recurrence, for disease-specific and overall survival in cases of advanced LSCC.

## Supporting Information

S1 Fig Cumulative risk of recurrence and death during 10 years' follow-up among patients with laryngeal squamous cell carcinoma. (DOCX)

S2 Fig Cumulative risk of recurrence by subsite of laryngeal squamous cell carcinoma during 10 years' follow-up. P value is achieved from the Pepe and Mori test comparing the cumulative risk of glottis versus supraglottic cancer.
(DOCX)
