# Supplementary material for: Risk of Recurrence in Laryngeal Cancer
Source: PLoS One. 2016 Oct 7;11(10):e0164068. doi: 10.1371/journal.pone.0164068 (PMC5055342; doi:10.1371/journal.pone.0164068)
Supplement: S1 Fig — (DOCX) [file pone.0164068.s001.docx]

**Supporting Information Figure 1**

**S1 Fig. 1: Cumulative risk of recurrence and death during 10 years' follow-up among patients with laryngeal squamous cell carcinoma.**
