# Supplementary material for: Risk of Recurrence in Laryngeal Cancer
Source: PLoS One. 2016 Oct 7;11(10):e0164068. doi: 10.1371/journal.pone.0164068 (PMC5055342; doi:10.1371/journal.pone.0164068)
Supplement: S2 Fig — P value is achieved from the Pepe and Mori test comparing the cumulative risk of glottis versus supraglottic cancer. (DOCX) [file pone.0164068.s002.docx]

**Supporting Information Figure 2**

**S2 Fig. 2: Cumulative risk of recurrence by subsite of laryngeal squamous cell carcinoma during 10 years' follow-up. P value is achieved from the Pepe and Mori test comparing the cumulative risk of glottic versus supraglottic cancer.**
